# A new scoring function for top-down spectral deconvolution

**DOI:** 10.1186/1471-2164-15-1140

**Published:** 2014-12-18

**Authors:** Qiang Kou, Si Wu, Xiaowen Liu

**Affiliations:** Department of BioHealth Informatics, Indiana University-Purdue University Indianapolis, 535 W. Michigan Street, Indianapolis, IN 46202 USA; Environmental Molecular Sciences Laboratory, Pacific Northwest National Laboratory, 902 Battelle Boulevard, Richland, WA 99352 USA; Center for Computational Biology and Bioinformatics, Indiana University School of Medicine, 410 West 10th Street, HS 5000, Indianapolis, IN 46202 USA

**Keywords:** Mass spectrometry, Deconvolution, Software

## Abstract

**Background:**

Top-down mass spectrometry plays an important role in intact protein identification and characterization. Top-down mass spectra are more complex than bottom-up mass spectra because they often contain many isotopomer envelopes from highly charged ions, which may overlap with one another. As a result, spectral deconvolution, which converts a complex top-down mass spectrum into a monoisotopic mass list, is a key step in top-down spectral interpretation.

**Results:**

In this paper, we propose a new scoring function, L-score, for evaluating isotopomer envelopes. By combining L-score with MS-Deconv, a new software tool, MS-Deconv+, was developed for top-down spectral deconvolution. Experimental results showed that MS-Deconv+ outperformed existing software tools in top-down spectral deconvolution.

**Conclusions:**

L-score shows high discriminative ability in identification of isotopomer envelopes. Using L-score, MS-Deconv+ reports many correct monoisotopic masses missed by other software tools, which are valuable for proteoform identification and characterization.

**Electronic supplementary material:**

The online version of this article (doi:10.1186/1471-2164-15-1140) contains supplementary material, which is available to authorized users.

## Background

In the last two decades, bottom-up mass spectrometry (MS) has been the mainstream of proteomics analysis [1–4]. Although it is efficient and high-throughput for protein identification and quantification, bottom-up MS has its limitations. It involves a sample preparation step in which long proteins are digested into short peptides by proteases, reducing its ability to identify various proteoforms with multiple changes, such as mutations, post-translational modifications (PTMs), and degradations [5, 6]. In contrast, top-down MS analyzes intact proteins, making it the method of choice for complex proteoform identification.

In a mass spectrum, each peak is represented as (*m*/*z*,*intensity*), where *m*/*z* and *intensity* are the mass-to-charge ratio and abundance of its corresponding ion, respectively. Because of the existence of natural isotopes, ions of the same chemical formula and charge state have different *m*/*z* values and correspond to a list of spectral peaks in a mass spectrum, called an *isotopomer envelope*. The *monoisotopic mass* of an ion is the sum of its atomic masses using the most abundant isotope for each of its atoms.

Compared with bottom-up mass spectra, top-down mass spectra are more complex because they often contain many high charge state isotopomer envelopes, some of which overlap with one another [7, 8]. As a result, a key step in top-down spectral interpretation is to deconvolute a complex top-down mass spectrum to a list of monoisotopic masses.

Given the chemical formula and charge state of an ion, its theoretical isotopomer distribution can be calculated based on the frequencies of natural isotopes. When the chemical formula is unknown and the only available information is its monoisotopic or average mass, the well-known averagine model [9] can be used to estimate the chemical formula from the monoisotopic or average mass. A theoretical isotopomer distribution is represented as a list of theoretical peaks (*m*/*z*,*probability*), in which *m*/*z* and *probility* are the mass-to-charge ratio and probability of the corresponding isotopomer. In top-down spectral deconvolution, theoretical isotopomer distributions are utilized to identify and group isotopic peaks.

Spectral deconvolution of profile mass spectra has been studied by several groups [7, 10]. In this paper, we focus on centroided spectra. While profile spectra keep all information of raw data, centroided spectra simplify data representation and speed up spectral deconvolution. Similar to mass spectra, isotopomer distributions can be represented in the profile or the centroided mode (Additional file 1: Figures S1 and S2). The centroided mode will be used in the proposed scoring function.

Many software tools have been developed for top-down spectral deconvolution [7, 10–12]. Most tools deconvolute a top-down mass spectrum in four steps. First, candidate isotopomer envelopes are extracted from the experimental spectrum and matched to theoretical isotopomer distributions. Second, the theoretical isotopomer distribution in a match is converted into a theoretical isotopomer envelope by scaling the probabilities to theoretical peak intensities. The scale ratio is estimated based on the peak intensities of the experimental isotopomer envelope. Third, the matches are evaluated by a scoring function, and a match is reported only if its score is higher than a specified threshold. Finally, a monoisotopic mass is obtained from each of the reported isotopomer envelopes.

The scoring function for evaluating experimental isotopomer envelopes determines the accuracy and sensitivity of spectral deconvolution. Designing a good scoring function is a challenging problem because complex mass spectra often contain many noise peaks and overlapping isotopomer envelopes. Most software tools use scoring functions based on the intensities of peaks in a pair of experimental and theoretical isotopomer envelopes, such as the sum of squared errors of peak intensities [7], the ratios of neighbouring peak intensities [13], and the dot product of intensity distributions [12]. The scoring function in MS-Deconv [8] combines peak intensities and errors in *m*/*z* values.

In this paper, we present a new scoring function, L-score, for computing the similarity between a pair of experimental and theoretical isotopomer envelopes. L-score can be used independently for spectral deconvolution or combined with other spectral deconvolution tools for envelope selection. We developed a software tool, MS-Deconv+, by combining MS-Deconv and L-score. Experiments showed that MS-Deconv+ outperformed other existing software tools in top-down spectral deconvolution.

## Methods

### Data sets

A data set from *Salmonella typhimurium* (ST) [14] was used for training and testing L-score. Cell lysate obtained from ST was analyzed with a C4-based high-performance liquid chromatography (HPLC) column coupled with an LTQ-Orbitrap mass spectrometer. A total of 4,636 collision induced dissociation (CID) tandem mass spectra were acquired. The charge states of the spectra range from 1 to 24; the precursor masses of the spectra range from 1,000 to 20,000 Dalton (Da). (See Ref. [14] for the detailed experimental procedure.)

Two *Escherichia coli* (EC) data sets were utilized to test L-score and MS-Deconv+. Cell lysate of EC was analyzed by a reversed phase liquid-chromatography (RPLC) coupled with an LTQ-Orbitrap Velos mass spectrometer. A total of 3,704 higher-energy C-trap dissociation (HCD) and 4,045 electron-transfer dissociation (ETD) tandem mass spectra were collected at a resolution of 60,000.

### Theoretical and experimental envelopes

Since the proposed scoring function is designed for centroided data, only centroided isotopomer distributions and centroided mass spectra are studied. In a centroided isotopomer distribution, two isotopomers are treated as the same if they contain the same number of neurons. For example, a water molecule with two ^1^H atoms and one ^18^O atom and another water molecule with two ^2^H atoms and one ^16^O atom are treated as the same although their masses are slightly different. As a result, isotopomers with the same number of neurons are represented by one peak. When the charge state of an ion is *z*, the distance between two neighbouring peaks in its centroided theoretical isotopomer distribution is approximately 1.00235/*z* thomson (Th) [7].

Top-down mass spectra contain some noise peaks. The noise intensity level of a spectrum is estimated by plotting the histogram of the peak intensity distribution and assuming that it is in the intensity bin with the largest number of peaks [7]. A peak is considered as a *signal peak* if its intensity is higher than the noise intensity level. In addition, a charge state is *valid* if it is no larger than a user-defined parameter.

**Figure 1:**
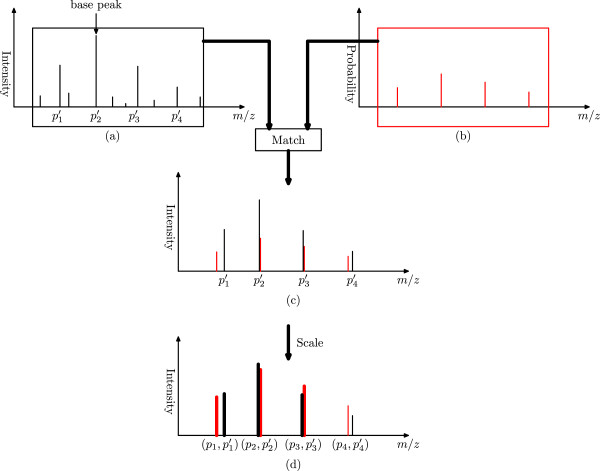
**Steps for matching a theoretical envelope with an experimental envelope.** **(a)** A mass spectrum contains an experimental envelope with four peaks *p*1′,*p*2′,*p*3′,*p*4′. One peak *p*2′ is selected as the base peak. **(b)** For a given charge state *z*, the averagine formula of a charge *z* ion is computed such that the *m*/*z* value of its most abundant isotopomer equals that of the base peak, and the theoretical isotopomer distribution is obtained. **(c)** The theoretical peaks in the theoretical isotopomer distribution are matched to experimental peaks with similar *m*/*z* values in the mass spectrum. **(d)** The theoretical peak intensities, which are initialized as the isotopomer probabilities, are scaled so that the sum of the intensities of the top three peaks (red bold peaks) in the theoretical envelope is the same to that (black bold peaks) of the experimental envelope.

We generate a theoretical isotopomer envelope as follows. First, we select a signal peak from a mass spectrum and a valid charge state *z*. The signal peak is called the *base peak* of the theoretical and its corresponding experimental isotopomer envelopes. Second, using the averagine model, we find a monoisotopic mass and its corresponding theoretical isotopomer distribution with the charge state *z* such that the *m*/*z* value of its most abundant isotopomer equals that of the base peak. Third, the peaks in the theoretical isotopomer distribution are matched to experimental peaks with similar *m*/*z* values in the spectrum. Finally, the intensities of the theoretical peaks are initialized as their probabilities and further scaled based on the intensities of the matched experimental peaks (Figure 1). Following the method in MS-Deconv, we scale theoretical peak intensities so that the sum of the intensities of the top three theoretical peaks equals that of their corresponding experimental peaks. If the scaled intensity of a theoretical peak is not higher than the noise intensity level, the theoretical peak is removed. The list of the remaining *scaled* peaks is referred to as a theoretical isotopomer envelope, or a *theoretical envelope* for brevity.

Given a theoretical envelope, a list of experimental peaks is extracted from the mass spectrum to form its corresponding *experimental envelope* by matching each peak in the theoretical envelope to an experimental peak with a similar *m*/*z* value (within an error tolerance). If such an experimental peak is not found, we add into the spectrum a zero-intensity peak whose *m*/*z* value is equal to the theoretical peak. A theoretical envelope and its matched experimental envelope are called an *envelope match*.

### Training and test data sets

We generated and annotated a set of envelope matches from the ST data set for training and testing L-score. In short, after tandem mass spectra were identified by database search, the resulting protein-spectrum-matches were utilized to obtain annotated envelope matches. The detailed steps are described below.

ReAdW (http://tools.proteomecenter.org/software.php) was used to convert the Thermo raw file into a centroided mzXML file. MS-Deconv [8] was applied to extract a list of monoisotopic masses and their corresponding envelope matches from each tandem mass spectrum of the ST data set. The deconvoluted mass lists were searched against a target-decoy concatenated ST proteome database using MS-Align+ [15]. Default parameter settings were used in MS-Deconv and MS-Align+. The Benjamini-Hochberg procedure [16, 17] was employed to estimate false discovery rates (FDRs) for identified protein-spectrum-matches. When the E-value cutoff was 5.74×10^-4^, a total of 493 target protein-spectrum-matches were identified and no decoy protein-spectrum-matches were reported. We assume the 493 target protein-spectrum-matches are all correct because they have an estimated 0*%* spectrum level FDR. Of the “correct” identifications, 468 protein-spectrum-matches, from 83 proteoforms of 67 proteins, do not contain PTMs (some may have truncations).

Since the PTM localization problem has not been fully solved in top-down spectral analysis, we used only the 468 protein-spectrum-matches without PTMs to generate training and test envelope matches. For each of the 83 proteoforms, we selected only one identified spectrum with the largest number of monoisotopic masses to remove similar spectra. The resulting 83 spectra contained 7,995 envelope matches. If the monoisotopic mass of an envelope match was mapped to a theoretical fragment ion of the identified proteoform within 15 parts per million (ppm), the envelope match was labeled as “correct”, otherwise, “incorrect”. Since the data set contains only CID tandem mass spectra, b- and y-ions as well as b- and y-ions with neutral losses (b-H _2_O, b-N _3_H, y-H _2_O, y-N _3_H) were used for labeling envelope matches. In addition, ±1 Da errors were allowed in mapping monoisotopic masses of envelopes to theoretical fragment ions because they are common in extracting monoisotopic masses from isotopomer envelopes. Out of the 7,995 envelope matches, 3,726 were labeled as “correct”, and 4,269 were labeled as “incorrect”.

L-score uses several features whose computation involves the number of peak pairs in an envelope match. Thus, we divided the 7,995 envelope matches into 4 groups with 2, 3, 4, and ≥5 peak pairs, which contained 924,1,284,2,017, and 3,770 envelope matches, respectively. The envelope matches in each group were randomly divided into training and test envelope matches of the same size. (If one group contains 2*n*+1 envelope matches, where *n* is an integer, the training data set contains *n* envelope matches and the test data set contains *n*+1 envelope matches).

We also generated a test set of envelope matches from the EC HCD data set. Following the method for the ST data set, we identified 1,537 protein-spectrum-matches with an estimated 0*%* spectrum level FDR, including 625 protein-spectrum-matches without PTMs from 242 proteoforms of 109 proteins. For each of the 242 proteoforms, we chose a matched spectrum with the largest number of monoisotopic masses. Finally, a set of 27,091 envelope matches was obtained, including 9,744 “correct” and 17,347 “incorrect” ones. They were further divided into 4 groups with 2,3,4, and ≥5 peak pairs, which contained 1,535, 4,572, 3,894, and 17,090 envelope matches, respectively.

### Features of envelope matches

Let *S* be an experimental mass spectrum. A peak in an isotopomer envelope is represented by a pair (*x*,*y*), where *x* and *y* are the *m*/*z* value and intensity, respectively. Let *E*=(*x*_1_,*y*_1_),(*x*_2_,*y*_2_),⋯, (*x*_*k*_,*y*_*k*_) be a theoretical envelope where *x*_1_<*x*_2_<…<*x*_*k*_, and *E*^′^=(*x*1′,*y*1′),(*x*2′,*y*_2_),⋯,(*xk*′,*yk*′) its corresponding experimental envelope in *S*. Each theoretical peak (*x*_*i*_,*y*_*i*_) is mapped to the experimental peak (*xi*′,*yi*′) for 1≤*i*≤*k*. Below we describe five features for distinguishing correct envelope matches from incorrect ones.

#### *M*/*z*values

In a correct experimental envelope, a peak is likely to have the same *m*/*z* value to its corresponding theoretical peak. Differences in *m*/*z* values between experimental and theoretical peaks are an effective feature for envelope evaluation. The squared *m*/*z* error between two peaks (*x*,*y*) and (*x*^′^,*y*^′^) is (*x*-*x*^′^)^2^ (Additional file 1: Figure S3). The *m*/*z* distance between *E* and *E*^′^ is the root mean square deviation of the *m*/*z* values of their matched peak pairs. If a theoretical peak does not match any experimental peak and a zero-intensity peak is added to form a peak pair, the peak pair is excluded from the computation of the *m*/*z* distance. Let *P* be the set of peak pairs of *E* and *E*^′^ without zero-intensity peaks. We define


#### Peak intensity distributions

The difference between the peak intensities of a theoretical peak and its corresponding experimental peak in correct envelope matches is often smaller than that in incorrect ones [7]. To design the distance function for peak intensities used in L-score, the following factors are considered. First, experimental envelopes have various average peak intensities. To compare these envelopes, raw peak intensities are converted into relative intensities by dividing them by the largest peak intensity in the theoretical envelope. For a peak with raw intensity *y*, the relative intensity of the peak is *r*(*y*)=*y*/*y*_*h*_, where *y*_*h*_ is the raw intensity of the highest peak in the theoretical envelope (Additional file 1: Figure S4). Second, a correct experimental peak may overlap with peaks from other envelopes, making its intensity error very large. To make the feature more reliable, a threshold is introduced so that the distance function is not significantly affected by one very large error in a pair of matched peaks. Third, the difference between the intensities of a theoretical peak (*x*,*y*) and its corresponding experimental peak (*x*^′^,*y*^′^) may be large, e.g., *r*(*y*^′^)-*r*(*y*)>0.5 or *r*(*y*^′^)-*r*(*y*)<-0.5. The main reason for the first case (*r*(*y*^′^)-*r*(*y*)>0.5) is that the experimental peak overlaps with other peaks, but the reason for the second case (*r*(*y*^′^)-*r*(*y*)<-0.5) is not clear. It is more frequent to observe the first case than the second (Additional file 1: Figure S5). Thus, a penalty factor is applied to the second case. Let *t* be the threshold for large errors and *c* the penalty factor. The distance function of a theoretical peak *p*=(*x*,*y*) and its corresponding experimental peak *p*^′^=(*x*^′^,*y*^′^) is


In the experiments, *t*=0.5 and *c*=2. The distance between the intensity distributions of *E* and *E*^′^ is the root mean square of the intensity distances of their matched peak pairs:


#### Supporting envelopes

In top-down spectral deconvolution, the first step is to extract from a mass spectrum a list of candidate experimental envelopes that satisfy some basic requirements [8]. For example, a candidate experimental envelope cannot have 3 or more missing peaks. If the candidate envelope list contains two envelopes that have the same monoisotopic mass and different charge states, then one envelope is called a *supporting envelope* of the other. For an experimental envelope *E*^′^ with *f* supporting envelopes, we define


#### Neutral loss envelopes

If the monoisotopic masses *m*_1_ and *m*_2_ of two envelopes *E*1′ and *E*2′ in the candidate envelope list satisfy that *m*_1_-*m*_2_ equals (within an error tolerance) the mass of an NH _3_ or H _2_O molecule, then *E*2′ is a neutral loss envelope of *E*1′. For an experimental envelope *E*^′^ with *f* neutral loss envelopes, we define


In the implementation of L-score, the envelope detection and selection methods in MS-Deconv are used to generate candidate envelope lists, in which supporting envelopes and neutral loss envelopes are identified.

#### Missing peak numbers

Peaks may be absent from experimental envelopes. In the generation of candidate envelopes, an experimental envelope is removed if it has ≥3 missing peaks, 3 theoretical peaks and 2 missing peaks, or 2 theoretical peaks and 1 missing peak. In addition, an experimental envelope is removed if it does not contain *k*-3 consecutive matched peaks, where *k* is the number of peaks in the theoretical envelope. As a result, most missing peaks in experimental envelopes are at the ends of isotopomer distributions. Therefore, locations of missing peaks are not included in L-score. Since envelope matches without missing peaks have a higher accuracy rate than those with missing peaks (Additional file 1: Figure S6), we introduce another feature *m*(*E*^′^) to represent the number of missing peaks in an experimental envelope *E*^′^.

### The scoring function

We designed L-score using a linear combination of the five features:


Logistic regression was applied to find the weights in the linear combination for each of the 4 groups (the peak pair number =2,3,4,≥5) using the ST training envelope matches. The resulting weights are listed in Table 1. The largest (absolute value) weight is from the feature of *m*/*z* distances, showing the importance of this feature.

**Table 1:** **The weights of the features in L-score reported by logistic regression using the ST training envelope matches**

Feature	#peak pairs =2		#peak pairs =3		#peak pairs =4		#peak pairs ≥5	
	Weight	P-value	Weight	P-value	Weight	P-value	Weight	P-value
M/z distance	3.237	3.5E-4	4.987	2.0E-16	3.942	2.0E-16	3.820	2.0E-16
Intensity distribution	0.851	0.028	1.565	2.8E-8	1.448	6.2E-8	1.349	2.0E-10
#supporting envelopes	-1.517	1.6E-5	-1.471	8.7E-9	-1.958	2.0E-16	-0.992	2.0E-16
#neutral loss envelopes	-2.491	5.5E-13	-0.810	2.38E-7	-0.795	2.8E-13	-0.343	1.1E-11
#missing peaks	-	-	0.277	0.198	0.386	9.1E-3	0.096	0.314

When the number of peak pairs is 2, the weight for the number of missing peaks (feature ***m(E*** ^***′***^ ***)***) is not used because this group does not contain any experimental envelopes with missing peaks.

To compare envelope matches from different peak pair number groups, we trained a lookup table for each peak pair number group to convert raw scores L(*E*,*E*^′^) to local FDRs using the ST training data set. Given a raw score and a peak pair number group, we count the numbers of correct and incorrect envelope matches in the training group whose scores are similar to the given score (the bin size is 0.02) and use the two numbers to estimate the local FDR. In practice, candidate envelope matches of a top-down mass spectrum are ranked and selected based on their estimated local FDRs.

### Combination of MS-Deconv and L-score

MS-Deconv deconvolutes top-down mass spectra with four steps. First, a list of envelope matches is generated by enumerating all valid charge states and all signal peaks in a mass spectrum as base peaks. Second, all envelope matches are filtered based on the number of missing peaks and the number of consecutive matched peaks. Third, a graph model is employed to select a small number of envelope matches from the list that can explain the spectrum well. Fourth, the number *x* of envelope matches to report is specified by the user or estimated by the precursor mass when a tandem mass spectrum is analyzed. (When the precursor mass is *M*, the length *L* of the target protein is estimated as ⌈*M*/*m*_avg_⌉, where *m*_avg_ is the average mass of the 20 amino acid residues, and the number of envelope matches to report is estimated as 2(*L*-1).) The envelope matches selected in the previous step are ranked by their similarity scores, and the top *x* envelope matches are reported. Finally, monoisotopic masses are extracted from the top *x* envelope matches. The similarity scoring function used in MS-Deconv is referred to as M-score.

To combine L-score with MS-Deconv, M-score is replaced by L-score (and the local FDR) in the fourth step of MS-Deconv (Additional file 1: Figure S7). By combining MS-Deconv and L-score, we developed a new software tool, MS-Deconv+, for top-down spectral deconvolution. Since local FDRs are reported with L-scores for envelope matches in MS-Deconv+, a local FDR threshold can be specified to decide the number of envelope matches to report.

In practice, MS-Deconv+ with default weights of features is first used to analyze data sets that are different from the training data set. To further improve the performance of MS-Deconv+, MS-Align+ can be utilized to identify highly confident protein-spectrum-matches and generate a set of training envelope matches to train the weights of features.

## Results and discussion

We implemented L-score and MS-Deconv+ in Java and tested them on the ST and EC data sets.

### Comparison of distance functions for peak intensity distributions

**Figure 2:**
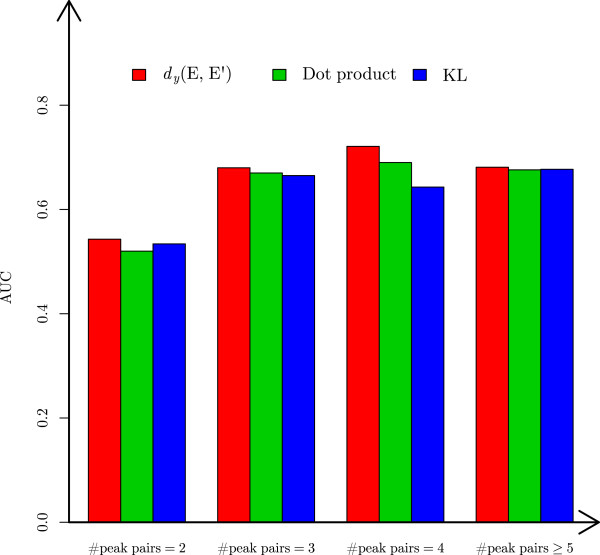
**Comparison of the distance function** ***d***_***y***_ **(** ***E*** **,** ***E*** ^***′***^ **), the dot product, and the KL divergence of peak intensity distributions on the ST test data set.** For each of the four groups (the number of peak pairs = 2, 3, 4, ≥5), the AUCs of the three functions are compared.

We proposed a function *d*_*y*_(*E*,*E*^′^) for measuring the distance between the peak intensity distributions of a theoretical envelope *E* and an experimental envelope *E*^′^. To evaluate the performance of the function, we compared it with the dot product and the Kullback-Leibler (KL) divergence of peak intensity distributions on the ST test envelope matches. The dot-product is a function for computing the similarity between two vectors, which is used in Hardklör [12]. In an envelope match (*E*,*E*^′^), the peak intensity distributions of the theoretical envelope *E*=(*x*_1_,*y*_1_),(*x*_2_,*y*_2_),…,(*x*_*k*_,*y*_*k*_) and the experimental envelope *E*^′^=(*x*1′,*y*1′),(*x*2′,*y*2′),…,(*xk*′,*yk*′) are represented as two vectors (*y*_1_,*y*_2_,…,*y*_*k*_) and (*y*1′,*y*2′,…,*yk*′). The two vectors are normalized to unit vectors before the dot product is calculated. The KL divergence is a function for measuring the relative entropy of one distribution from another distribution. For two discrete probability distributions *P* and *Q*, the KL divergence of *Q* from *P* is . To compute the KL divergence of *E*^′^ from *E*, the two vectors (*y*_1_,*y*_2_,…,*y*_*k*_) and (*y*1′,*y*2′,…,*yk*′) are converted into two probability distributions by dividing each peak intensity by the sum of peak intensities of the envelope. The three functions were tested on the 4 groups (the peak pair number =2,3,4,≥5) of the ST test envelope matches and compared based on the area under the curve (AUC) with respect to the receiver operating characteristic (ROC). The comparison shows that *d*_*y*_(*E*,*E*^′^) is more powerful than the other two functions in discriminating correct envelope matches from incorrect ones, especially when the envelopes contain 4 peak pairs (Figure 2).

### Discriminative abilities of single features and L-score

**Figure 3:**
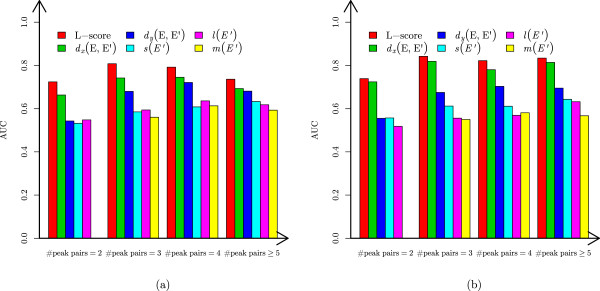
**Comparison of L-score and the five single features.** The AUCs of L-score and the five single features for the four groups (the number of peak pairs =2,3,4,≥5) are compared. **(a)** The ST data set. **(b)** The EC HCD data set.

We tested the discriminative abilities of the five single features and L-score on the ST test data set and the EC HCD test data set (Figure 3). The *m*/*z* distance has the best AUC among all the features. Compared with the single features, L-score improves the discriminative ability, demonstrating the advantage of combining multiple features (Figure 3).

**Figure 4:**
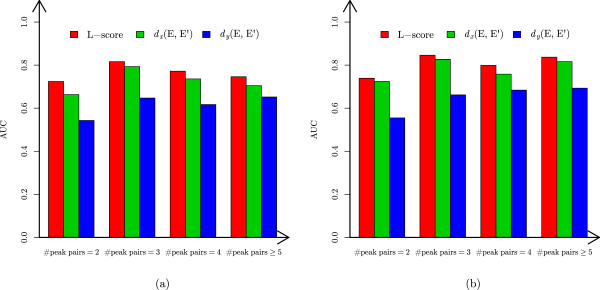
**Comparison of L-score and the two features for** ***m*** **/** ***z*** **distances and peak intensity distributions on envelope matches without missing peaks.** The AUCs of L-score and the two single features for the four groups (the number of peak pairs =2,3,4,≥5) are compared. **(a)** The ST data set. **(b)** The EC HCD data set.

Some test envelope matches have missing peaks, but the features for *m*/*z* distances and peak intensity distributions do not utilize this important information. We further compared the performance of the two features and L-score on envelope matches without missing peaks (Figure 4). L-score still outperformed the two single features in evaluating envelope matches.

### Comparison with other scoring functions

**Figure 5:**
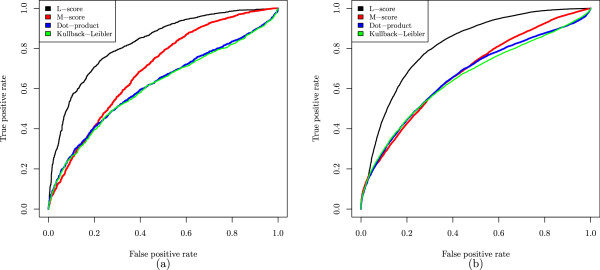
**Comparison of the ROC curves of L-score, M-score, the dot product, and the KL divergence on the ST and EC HCD test data sets.** **(a)** The ST test data set. The AUCs of L-score, M-score, the dot product, and the KL divergence are 0.825, 0.696, 0.608, and 0.602, respectively. **(b)** The EC HCD test data set. The AUCs of L-score, M-score, the dot product, and the KL divergence are 0.816, 0.678, 0.663, and 0.655, respectively.

We compared L-score with M-score, the dot product, and the KL divergence on the 3,998 envelope matches in the test ST data set and the 16,020 envelope matches in the test EC ETD data set. (See Additional file 1 for the parameter settings.) The ROC curves of the four functions demonstrate that M-score and L-score significantly increase the AUC compared with the other two functions (Figure 5). Compared with M-score, L-score increases the AUC from 0.696 to 0.825 for the ST test envelope matches and from 0.678 to 0.816 for the EC HCD test envelope matches.

### Combination of L-score and Decon2LS

Decon2LS [11], a reimplementation of THRASH [7], reports a list of ranked envelope matches from a top-down mass spectrum. To test L-score coupled with Decon2LS, L-score was utilized to re-rank the envelope matches in the list reported by Decon2LS. Two lists of ranked envelope matches (one by Decon2LS and the other by L-score coupled with Decon2LS) were generated for each of 242 mass spectra in the EC HCD test data set. For each *i*=1,2,…,20, we collected two sets of envelope matches with the rank *i* from the lists of ranked envelope matches reported by Decon2LS and L-score coupled with Decon2LS and then compared their accuracy rates (Additional file 1: Figure S8). L-score coupled with Decon2LS reported more correct top ranked enveloped matches than Decon2LS. In practice, when Decon2LS reports *x* envelope matches from a mass spectrum, the following procedure can be used to boost the accuracy rate of reported envelope matches. The RL-value threshold of Decon2LS is lowered so that the number of envelope matches extracted from the spectrum is larger than *x*. Then L-score is utilized to re-rank the envelope matches, and only the top *x* ones are reported.

### Comparison of Decon2LS, MS-Deconv and MS-Deconv+ on spectral identification

**Figure 6:**
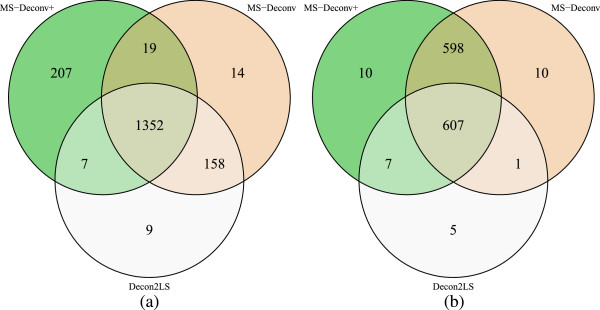
**Comparison of Decon2LS, MS-Deconv, and MS-Deconv+ on spectral identification by coupling them with MS-Align+.** The numbers of tandem mass spectra identified from the EC HCD and ETD data sets by the three methods with 1% protein level FDR are compared. **(a)** The EC HCD data set. **(b)** The EC ETD data set.

All the tandem mass spectra in the EC HCD and ETD data sets were deconvoluted by Decon2LS, MS-Deconv, and MS-Deconv+; the deconvoluted mass lists reported by the three tools were searched against the EC proteome for protein identification using MS-Align+ [15]. (See Additional file 1 for the parameter settings of MS-Align+ and the three tools.) The EC proteome database was downloaded from the Swiss-Prot database, and a shuffled database of the same size was concatenated to the target protein database for estimation of FDRs. With 1*%* protein level FDR, MS-Deconv+ coupled with MS-Align+ identified more spectra (1,585 in HCD and 1,223 in ETD) than MS-Deconv (1,543 in HCD and 1,216 in ETD) and Decon2LS (1,526 in HCD and 620 in ETD) (Figure 6). The three methods shared a total of 1,352 spectral identifications in the EC HCD data set and 607 spectral identifications in the EC ETD data set. Although the performances of MS-Deconv+ and MS-Deconv were similar in the number of identified spectra, MS-Deconv+ reported more matched monoisotopic masses (55,731 in HCD and 24,235 in ETD) than MS-Deconv (41079 in HCD and 21,360 in ETD) and Decon2LS (39,991 in HCD and 10,479 in ETD) for the spectra identified by all the tools. These matched masses play an important role in localizing various changes in identified proteoforms.

## Conclusions

In this paper, we proposed L-score for evaluating experimental isotopomer envelopes, which outperformed existing scoring functions in distinguishing correct experimentalenvelopes from incorrect ones. We further developed MS-Deconv+, a top-down spectral deconvolution tool that combines MS-Deconv and L-score. In the experiments on the two EC data sets, MS-Deconv+ reported more correct monoisotopic masses than MS-Deconv. These correct monoisotopic masses provide essential information for proteoform identification and characterization.

## Declarations

Publication of this article was funded by a startup fund provided by Indiana University-Purdue University Indianapolis.

## Electronic supplementary material

Additional file 1: **Supplementary material.** (PDF 139 KB)
