# Supplementary material for: A new scoring function for top-down spectral deconvolution
Source: BMC Genomics. 2014 Dec 18;15(1):1140. doi: 10.1186/1471-2164-15-1140 (PMC4378558; doi:10.1186/1471-2164-15-1140)
Supplement: Supplementary file 1 — Additional file 1: Supplementary material. (PDF 139 KB) [file 12864_2014_6824_MOESM1_ESM.pdf]

# A new scoring function for top-down spectral deconvolution

# (Supplementary Material)

## 1 The profile and centroided modes for isotopomer distributions

The profile and centroided modes for representing isotopomer distributions are shown in Figures S1 and S2.

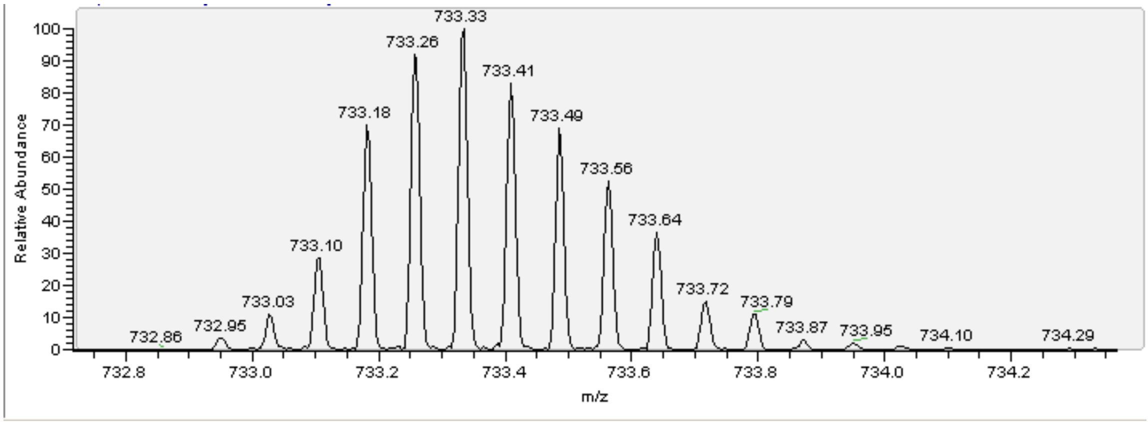


Figure S1: An example for the profile mode representation of isotopomer distributions.

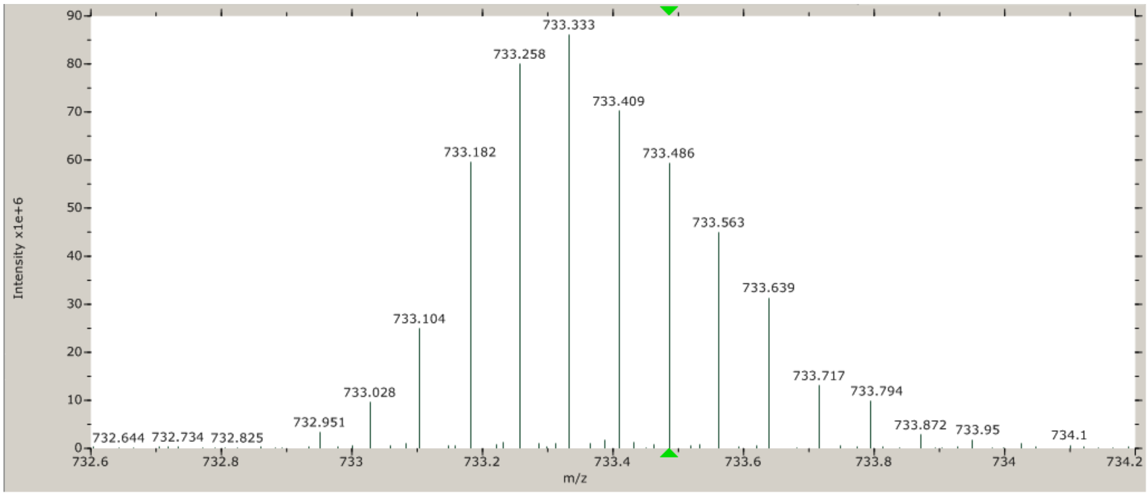


Figure S2: An example for the centroided mode representation of isotopomer distributions.

## 2 M/z errors of peak pairs

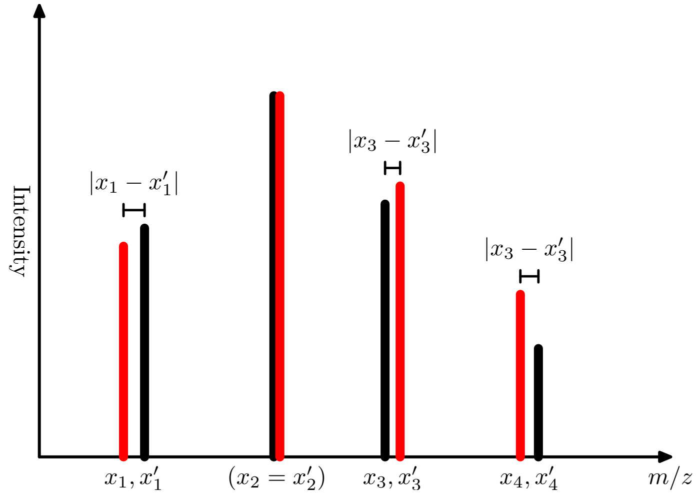


Figure S3: The $m/z$ errors of peak pairs between a theoretical envelope $E = (x_1, y_1), (x_2, y_2), (x_3, y_3), (x_4, y_4)$ and an experimental envelope $E' = (x_1', y_1'), (x_2', y_2'), (x_3', y_3'), (x_4', y_4')$, in which $x_2 = x_2'$.

# 3 Intensity distances of peak pairs

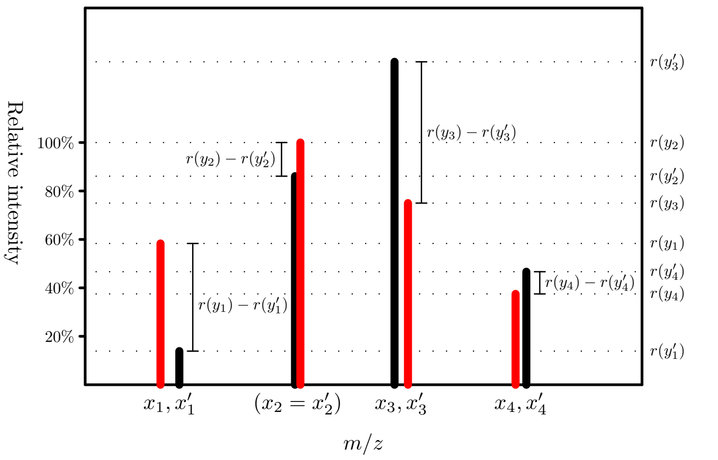


Figure S4: The intensity distances of peak pairs between a theoretical envelope $E = (x_1, y_1), (x_2, y_2), (x_3, y_3), (x_4, y_4)$ and an experimental envelope $E' = (x_1', y_1'), (x_2', y_2'), (x_3', y_3'), (x_4', y_4')$. A raw intensity $y$ is converted into a relative intensity $r(y) = y/y_h$, where $y_h$ ($y_h = y_2$ in the example) is the highest peak intensity in the theoretical envelope.

# 4 The distribution of the intensity differences between theoretical and experimental peaks in envelope matches

For each theoretical peak $p = (x, y)$ and its matched experimental peak $p' = (x', y')$ in the $1,998$ ST training envelope matches, we converted intensities $y$ and $y'$ into relative intensities $r(y) = y/y_h$ and $r(y') = y'/y_h$, where $y_h$ is the intensity of the highest peak in the theoretical envelope. The histogram of $r(y') - r(y)$ shows that it is more frequent to observe $r(y') - r(y) > 0.5$ than $r(y') - r(y) < -0.5$ (Figure S5).

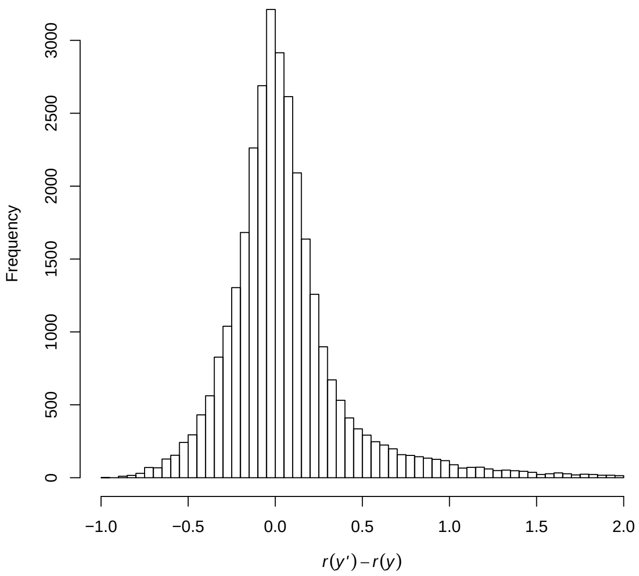


Figure S5: The histogram of $r(y') - r(y)$ generated from the ST training envelope matches.

# 5 Relationship between the number of missing peaks and the accuracy rate of envelope matches

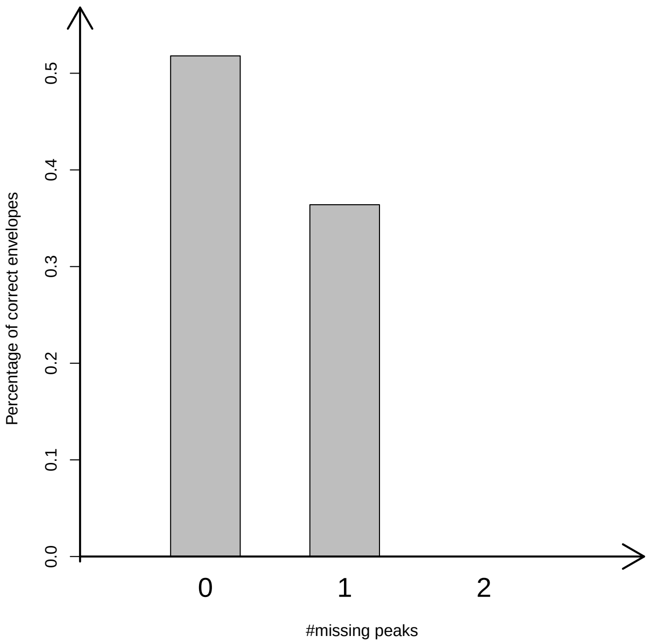


Figure S6: The accuracy rates of envelope matches with 0, 1, and 2 missing peaks in the ST training data set. The accuracy rate for envelope matches with 2 missing peaks is 0%.

# 6 Combination of MS-Deconv and L-score

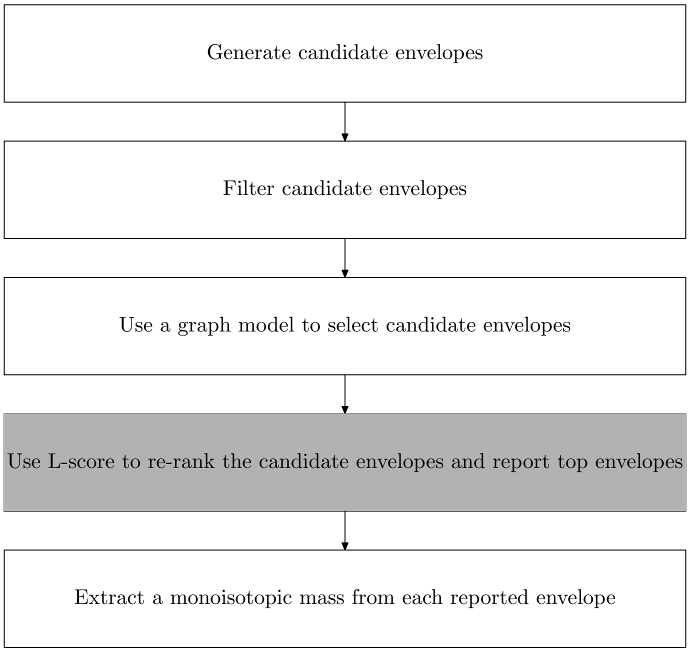


Figure S7: The main steps in MS-Deconv+. To combine MS-Deconv+ and L-score, the scoring function in MS-Deconv is replaced by L-score in the fourth step.

# 7 Parameter settings in Decon2LS

We used all default parameter settings in Decon2LS except for the 3 parameters listed in Table S1.

Table S1: Parameter settings in Decon2LS

| Parameter | Value |
|---|---|
| MaxFit | 0.7 |
| ProcessMSMS | True |
| Process_MS | False |

# 8 Parameter settings in MS-Deconv and MS-Deconv+

Table S2: Parameter settings in MS-Deconv and MS-Deconv+

| Parameter | Value |
|---|---|
| Maximum charge | 30 |
| Maximum mass | 50000.0 |
| m/z tolerance | 0.02 |
| s/n ration | 1.0 |
| Report MS1 | false |

# 9 Parameter settings in MS-align+

Table S3: Parameter settings in MS-align+

| Parameter | Value |
|---|---|
| searchType | TARGET+DECOY |
| activation | FILE |
| cysteineProtection | C0 |
| shiftNumber | 2 |
| errorTolerance | 15 |
| cutoffType | FDR |
| cutoff | 0.01 |
| doOneDaltonCorrection | false |
| doChargeCorrection | false |

## 10 Combination of Decon2LS and L-score

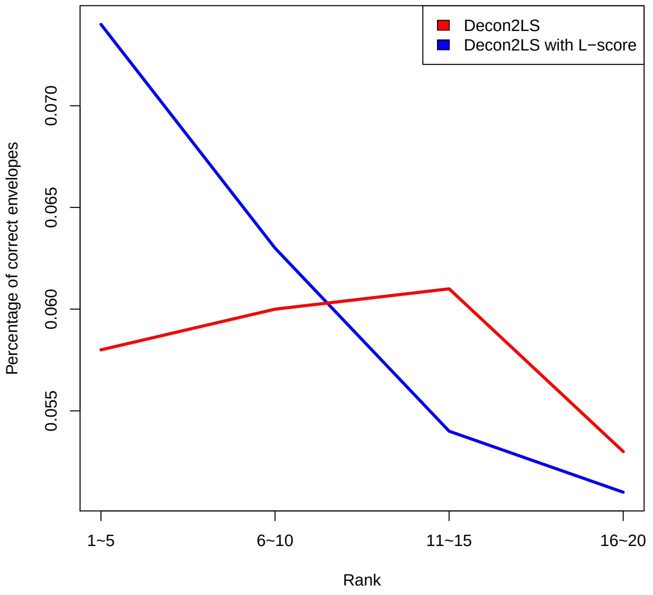


Figure S8: The accuracy rates of envelope matches in the ranked envelope match lists reported by Decon2LS alone and Decon2LS coupled with L-score on the EC HCD test data set.
